# Plants and Microbes Mediate the Shift in Ecosystem Multifunctionality From Low to High Patterns Across Alpine Grasslands on the Tibetan Plateau

**DOI:** 10.3389/fpls.2021.760599

**Published:** 2021-10-15

**Authors:** Yi Wang, Miao Liu, Youchao Chen, Tao Zeng, Xuyang Lu, Bin Yang, Yafeng Wang, Lin Zhang, Xiaowei Nie, Feipeng Xiao, Zhigang Zhang, Jian Sun

**Affiliations:** ^1^College of Management Science and Engineering, Guangxi University of Finance and Economics, Nanning, China; ^2^School of Life Sciences and School of Ecology, State Key Laboratory of Biological Control, Sun Yat-sen University, Guangzhou, China; ^3^State Key Laboratory of Tibetan Plateau Earth System, Resources and Environment (TPESRE), Institute of Tibetan Plateau Research, Chinese Academy of Sciences, Beijing, China; ^4^College of Grassland Science and Technology, China Agricultural University, Beijing, China; ^5^Key Laboratory of Aquatic Botany and Watershed Ecology, Wuhan Botanical Garden, Chinese Academy of Sciences, Wuhan, China; ^6^College of Earth Sciences, Chengdu University of Technology, Chengdu, China; ^7^Key Laboratory of Mountain Surface Processes and Ecological Regulation, Institute of Mountain Hazards and Environment, Chinese Academy of Sciences, Chengdu, China; ^8^Natural Resources Comprehensive Survey Command Center, China Geological Survey, Beijing, China

**Keywords:** ecosystem multifunctionality, alpine grassland, plant community, microbial biomass, Tibetan Plateau

## Abstract

Both plant communities and soil microbes have been reported to be correlated with ecosystem multifunctionality (EMF) in terrestrial ecosystems. However, the process and mechanism of aboveground and belowground communities on different EMF patterns are not clear. In order to explore different response patterns and mechanisms of EMF, we divided EMF into low (<0) and high patterns (>0). We found that there were contrasting patterns of low and high EMF in the alpine grassland ecosystem on the Tibetan Plateau. Specifically, compared with low EMF, environmental factors showed higher sensitivity to high EMF. Soil properties are critical factors that mediate the impact of community functions on low EMF based on the change of partial correlation coefficients from 0 to 0.24. In addition, plant community functions and microbial biomass may mediate the shift of EMF from low to high patterns through the driving role of climate across the alpine grassland ecosystem. Our findings will be vital to clarify the mechanism for the stability properties of grassland communities and ecosystems under ongoing and future climate change.

## Introduction

Recently, due to the reduction of global biodiversity, researchers have paid considerable attention to the linkages between biodiversity and ecosystem functioning (Maestre et al., 2012; Wang et al., 2020a). Hence, ecosystem multifunctionality (EMF), which represents the simultaneous provision of multiple ecosystem functions, such as productivity, carbon storage, and the accumulation of nutrients, has been proposed as a reliable indicator to reflect the effects of ecosystem services and functioning (Xu et al., 2013; Jing et al., 2015). Currently, plant biodiversity has been considered to play a key role in regulating the performance of ecosystems (Hector and Hooper, 2002; Hector and Bagchi, 2007; Zavaleta et al., 2010; Sun et al., 2020a). For example, compared with low plant community diversity, higher plant community diversity could maintain higher soil moisture, which may restrain the adverse effects of drought or warming on soil microbes and retain high EMF (Milcu et al., 2010; Zavaleta et al., 2010). But taking grassland EMF patterns seriously is far from straightforward due to the unknown of the belowground community.

Soil microbial communities drive most biogeochemical processes and play a vital role in driving the cycling of carbon and nitrogen and their interactions (Chapin et al., 2008; Xu et al., 2013; Zhang et al., 2021). In general, an increased substrate for soil microbes results in more microbial biomass and a more abundant microbial community (Eisenhauer et al., 2010). The amount of microbial biomass carbon and nitrogen plays a major role in regulating the process of decomposition and sequestration of soil carbon, as well as nitrogen immobilization, which may further affect EMF in terrestrial ecosystems (Lange et al., 2015). However, most studies examining the EMF come from single investigations at plot scale (Tilman et al., 1997; Hillebrand and Matthiessen, 2009; Mori et al., 2016; Gross et al., 2017). Few studies have examined the influences of soil microbes on the patterns of EMF at a large regional scale due to high spatial heterogeneities in soil microbial properties and limitations of sampling and measuring methods (Joergensen et al., 2011; Schmidt et al., 2011).

Grasslands, covering approximately 40% of the Earth’s land, are sensitive to climate change and anthropogenic disturbance (Tilman et al., 2006; Reid et al., 2014), which could lead to a loss of above- and belowground biodiversity and a reduction in EMF (Hector and Bagchi, 2007; Mcsherry and Ritchie, 2013; Zhou et al., 2017). Previous studies have highlighted the effects of climate (Jing et al., 2015), plant communities (Diaz et al., 2007; Jing et al., 2015), soil microbes (Bonkowski and Roy, 2005), and mycorrhizal fungi (van der Heijden et al., 1998; Maherali and Klironomos, 2007) on the EMF. For abiotic drivers, several studies have shown that climate change and anthropogenic activities reduce the microbial abundance and the overall diversity of soil organisms (Helgason et al., 1998; De Vries et al., 2013; Wagg et al., 2014; Wang et al., 2019) and have concluded that the reduced biodiversity in soils may influence the EMF by impairing ecosystem functions such as the cycling of resources between above- and belowground communities (van der Heijden et al., 2008; Wall et al., 2010). Academics have revealed that precipitation is the main factor affecting the dynamics of vegetation in alpine grasslands, especially in arid areas (Hu et al., 2010), which may further regulate the ecosystem process and EMF. Additionally, the air temperature may be another driver in environmental geochemistry cycles and soil nutrient availability for plant growth (Zhou et al., 2020), which, to some extent, may contribute to EMF. For biotic drivers, sustaining EMF in grassland ecosystems requires both biodiversity assemblages and higher species richness (Zavaleta et al., 2010). In contrast, high EMF may require suitable environmental conditions. In addition, the complex interactions between soil factors and complex food webs can shape diversity within one trophic group or may change the microbial abundance, diversity, and EMF (Hunt and Wall, 2002; Duffy et al., 2007). However, most of these studies are more concerned that high above- and belowground biodiversity and fertile habitats may lead to high EMF. It is still far from clear how plant and soil microbial factors work better at different EMF levels, especially which factors critically mediate the transformation from lower to higher EMF across grassland ecosystems.

**Figure 1:**
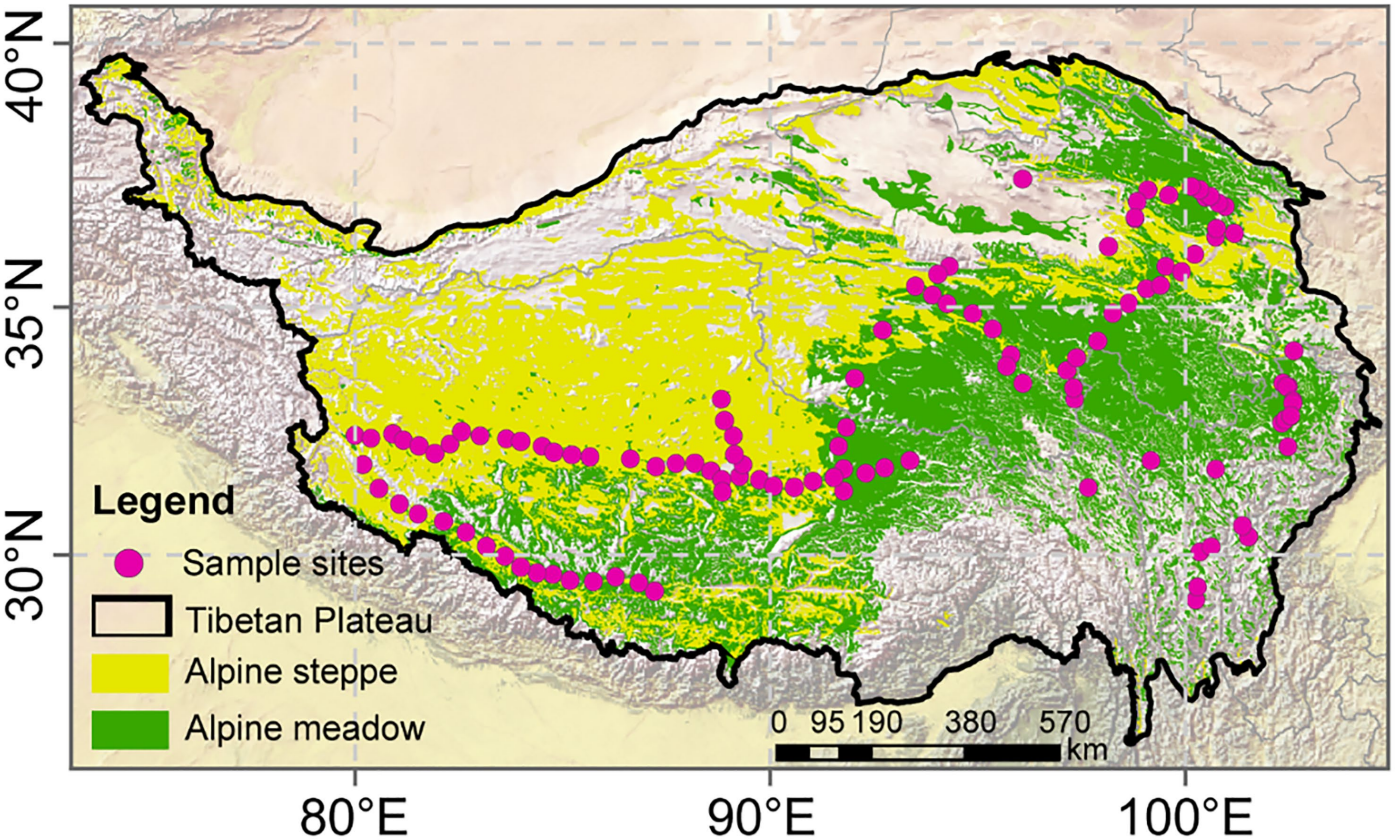
Vegetation map of the Tibetan Plateau grasslands and location of sampling sites in 2015.

To fill these knowledge gaps, we selected alpine grasslands on the Tibetan Plateau as the study area and analyzed data from 115 sites distributed along an extensive water–heat gradient (Figure 1). The Tibetan Plateau is the highest and most extensive highland on Earth with an extraordinarily sensitive ecosystem about alpine grasslands to climate change (Sun et al., 2020b). Particularly, the plateau has natural water–heat gradients from southeast to northwest, making it an ideal region to explore EMF patterns along with climate gradients (Yang et al., 2010a). Under the influence of hydrothermal patterns, different alpine grassland types are formed as alpine meadow, steppe, and desert steppe, and plants may lead to a series of differences in habitat conditions and community characteristics in the process of long-term adaptation to climatic conditions (Zhou et al., 2020). Therefore, under the climate gradient, a suite of adaptation and regulation processes of above- and belowground communities will eventually lead to the difference of EMF. Besides, based on these special environmental conditions, more comprehensive climate, plant, and soil factors were involved in our study to investigate the interacting effects of changing climate and ecosystem functions on the different levels of EMF. Hence, we aimed to examine how climatic factors, plant communities, soil properties, and microbial factors influenced EMF, and explore: (1) the different response patterns of EMF to environmental factors, and (2) highlight the underlying mechanisms of plants and microbes mediate the EMF patterns.

## Materials and Methods

### Study Area and Sampling

We collected plant and soil samples from 115 sites over a wide area (2.3×10^6^km^2^) on the Tibetan Plateau (80–105°E, 27–37°N; Figure 1) during the growing season of 2015. The sites covered two main vegetation types on the plateau, i.e., alpine meadow and alpine steppe. Random plots (10m×10m) were set up within an area with less human disturbance. Plant and soil samples were taken across a large area along a transect running from the northwest region of Sichuan Province (32°52'N, 102°25'E), to mid-Qinghai Province (34°52'N, 98°2'E), and finally to northern Tibet (33°24'N, 88°34'E) for the obvious water–heat gradients. The sites ranged in elevation from 3,030 to 5,000m.

### Plant Measurement

Before soil sampling, we counted the numbers of species and calculated the relative abundance of each species. Species richness (SR) was defined according to the number of species. We harvested all standing plants in three quadrats (50cm×50cm) to measure the aboveground biomass (AGB). Then, we ground part of the aboveground plant to a fine powder on a ball mill and analyzed plant nutrients. In addition, we collected 1,035 soil samples (0–10, 10–20, and 20–30cm; 3 samples per site) from the sites for the determination of soil physicochemical properties, and the sum of the root biomass at each depth was treated as the belowground biomass (BGB). The biomass samples were dried at 65°C to constant mass and weighed to the nearest 0.01g in the laboratory. We collected four plant traits: plant height (H), leaf nitrogen content (LN), leaf phosphorus content (LP), and leaf dry matter content (LDMC), which are known to be the key factors for acquiring sources and determining species abundance in grasslands (Ansquer et al., 2009). The plant height was calculated as the mean value of random individuals. We quantified functional diversity using community-weighted mean (CWM) values (Diaz et al., 2007), which were calculated as:


(1) $$CWM=\sum p_{i}\times trait_{i}$$

where *p_i_* is the relative abundance of species *i* in the community and *trait_i_* represents the trait value of species *i* (Lavorel et al., 2007).

### Physiochemical Data Measurements

For plant nutrients, LN was determined by combustion on a Vario MACRO cube elemental analyzer (Elementar Analysensysteme Gmb H, Germany; Lu et al., 2015). LP was measured with the molybdate colorimetric test (Sommers and Nelson, 1972). For soil properties, soil water content (SWC) and soil bulk density (SBD) were tested using a drying method (Yao, 2006); soil organic carbon content (SOC) and soil total nitrogen content (STN) were determined by combustion on a Vario MACRO cube elemental analyzer (Lu et al., 2015); and soil total phosphorus content (STP) was measured with the molybdate colorimetric test after perchloric acid digestion (Sommers and Nelson, 1972). Soil available nitrogen content (SAN) was determined from the alkali hydrolyzable fraction, and soil available phosphorus content (SAP) was measured by the Olsen method (Sun et al., 2018). For soil microbial factors, the determination of microbial biomass carbon content (MBC) and nitrogen content (MBN) was performed with the chloroform–K_2_SO_4_ extraction method (Vance et al., 1987).

### Climate and Phenological Data

For each of the sites, we compiled annual mean temperature (AMT) and annual total precipitation (ATP) from the National Meteorological Bureau of China database.^1^ The ATP and AMT raster databases at a resolution of 1km were spatially interpolated by Anusplin 4.2 (Centre for Resource and 220 Environmental Studies, Australian National University, Canberra). Our sites in AMT ranged from −2.4 to 6.8°C, and those in ATP ranged from 143 to 860mm. The aridity index (AI) was calculated according to the method of Sun et al. (2020b). Phenological data stemming from the Global Inventory Modelling and Mapping Studies (Gonsamo et al., 2018), phenology features of the start of the growing season, and the peak of the growing season in 2015 were determined through simple linear regression analysis. Then, the start of the growing season and the peak of the growing season were calculated using the derivatives of the fitted Fourier model (Sun et al., 2020b). Ultimately, the length of growing season accumulation (GSL) was calculated by the difference between the above two parameters.

### Ecosystem Multifunctionality

Several variables were used to assess ecosystem functions by plant nutrients: (1) LN, (2) LP; plant productivity: (3) AGB, (4) BGB; soil properties: (5) SWC, (6) SOC, (7) STN, (8) STP, (9) SAN, (10) SAP; microbe: (11) MBC, (12) MBN. Considering the feature direct and interpretable measure to synthesize the multiple functions simultaneously, we adopted the average approach and used the EMF index (Maestre et al., 2012).


(2) $$EMF=\sum_{j}^{n}Z_{ij}/n$$

*Z_ij_* is the Z-score of ecosystem function *j* of plot *i*, and *n* is the number of ecosystem functions. We calculated the Z-scores for each of the ecosystem functions evaluated (Maestre et al., 2012):


(3) $$Z_{ij}=\left(x_{ij}-\lambda_{j}\right)/\delta_{j}$$

*x_ij_* is the value of ecosystem function *j* of plot *i*, *λ_j_* is the mean value of ecosystem function *j* across all sites, and *δ_j_* is the standard deviation of ecosystem function *j* across all sites. Notably, in this study, EMF indices above 0 are defined as high EMF, and those below 0 are defined as low EMF.

### Statistical Analyses

First, we evaluated the relationships between effect factors and EMF using SPSS version 25.0 (SPSS, Inc., Chicago, IL, USA). Meanwhile, we conducted the box figure connected with the difference of EMF and environmental factors with Analysis of Variances, performed using the “*ggplot2*” and “*aov*” function in the base package of R version 3.6.0 (R Development Core Team). Second, a general linear model was utilized to explore the relationship of environmental factors and EMF under low and high EMF conditions by the packages “*ggplot2*” in R software. Partial correlations were adopted to judge main control elements by the packages “*plspm*,” “*Hmisc*,” “*ppcor*,” and “*igraph*” in R software. Ultimately, we used the structural equation model (SEM) to determine the direct and indirect effects of climate, productivity, community functions, and soil microbes on EMF, which includes a synthesis of factor analysis, path analysis, and maximum likelihood analysis (Doncaster, 2007). We eliminated nonsignificant pathways and state variables and simplified the primary model based on regression weight estimates. Each model was fitted with the root mean square error of approximation (RMSEA; the model has an acceptable fit when 0.00≤RMSEA ≤0.05). Moreover, the Akaike information criterion, a criterion for the goodness of fit of SEM, was also used to select the most appropriate model. All the models were developed with AMOS 21 software (IBM SPSS Inc.).

## Results

### Patterns of EMF

**Figure 2:**
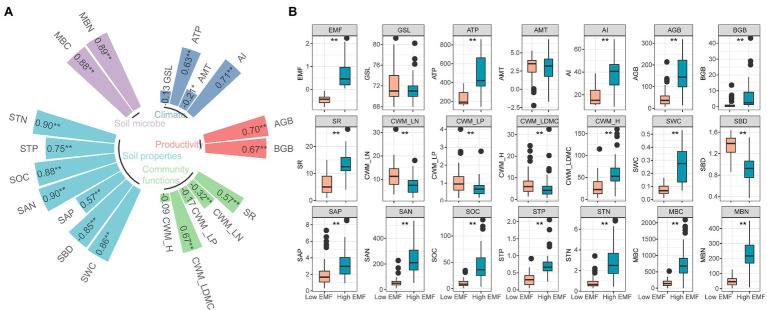
Characteristics of ecosystem multifunctionality and environmental factors. **(A)** Relationship among ecosystem multifunctionality and climate, productivity, community functions, soil properties, soil microbial factors, and the value in the square represents the value of the correlation coefficient between each factor with ecosystem multifunctionality. ^*^ and ^**^ show the significant correlations at 0.05 and 0.01 levels. **(B)** Difference of low and high ecosystem multifunctionality combined with environmental factors. Statistical significance between low and high ecosystem multifunctionality conditions was assessed by one-way ANOVA analysis. **p<0.01. EMF, ecosystem multifunctionality; GSL, start of growing season to the peak of growing season; ATP, annual total precipitation (mm); AMT, annual mean temperature (°C); AI, aridity index; AGB, aboveground biomass (gm^−2^); BGB, belowground biomass (gm^−2^); SR, species richness; CWM_LN, community-weighted mean value of leaf nitrogen; CWM_LP, community-weighted mean value of leaf phosphorus; CWM_LDMC, community-weighted mean value of leaf dry matter content; CWM_H, community-weighted mean value of plant height; SWC, soil water content (%); SBD, soil bulk density (gcm^−3^); SAP, soil available phosphorus content (mgkg^−1^); SAN, soil available nitrogen content (mgkg^−1^); SOC, soil organic carbon content (gkg^−1^); STP, soil total phosphorus content (%); MBC, microbial biomass carbon content (mgkg^−1^); MBN, microbial biomass nitrogen content (mgkg^−1^).

We found a high correlation coefficient of SAN (*r*=0.90), STN (*r*=0.90), MBN (*r*=0.89), MBC (*r*=0.88), SOC (*r*=0.88), and SWC (*r*=0.86) with EMF (*p*<0.01; Figure 2A). Besides, EMF showed significantly negative correlation with SBD (*r*=−0.86, *p*<0.01), CWM_LN (*r*=−0.32, *p*<0.01), and AMT (*r*=−0.21, *p*<0.05; Figure 2A). There was a significant difference between low EMF (mean value −0.44) and high EMF (mean value 0.66; *p*<0.01; Figure 2B). Except GSL and AMT, all environmental factors showed significant difference (*p*<0.01) under low EMF and high EMF conditions (Figure 2B).

### Different Response of EMF to Environmental Factors

**Figure 3:**
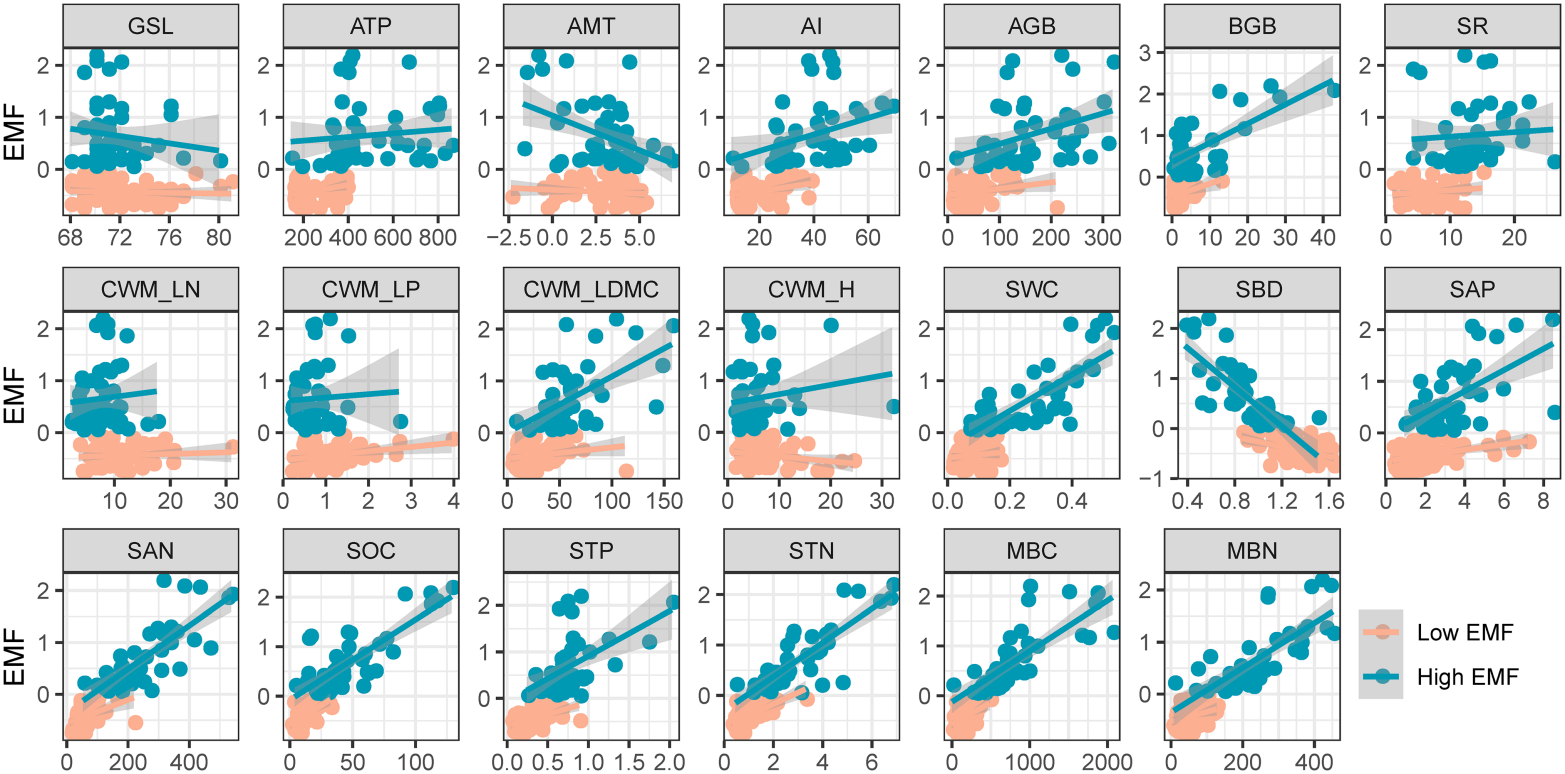
Relationship among low and high ecosystem multifunctionality and effect factors. EMF, ecosystem multifunctionality; GSL, start of growing season to the peak of growing season; ATP, annual total precipitation (mm); AMT, annual mean temperature (°C); AI, aridity index; AGB, aboveground biomass (gm^−2^); BGB, belowground biomass (gm^−2^); SR, species richness; CWM_LN, community-weighted mean value of leaf nitrogen; CWM_LP, community-weighted mean value of leaf phosphorus; CWM_LDMC, community-weighted mean value of leaf dry matter content; CWM_H, community-weighted mean value of plant height; SWC, soil water content (%); SBD, soil bulk density (gcm^−3^); SAP, soil available phosphorus content (mgkg^−1^); SAN, soil available nitrogen content (mgkg^−1^); SOC, soil organic carbon content (gkg^−1^); STP, soil total phosphorus content (%); MBC, microbial biomass carbon content (mgkg^−1^); MBN, microbial biomass nitrogen content (mgkg^−1^). Red and green lines are the fitted lines from ordinary least squares regressions. Shaded areas show the 95% CI of the fit.

By the fitted lines between EMF and effect factors, eight variables, including AI, AGB, BGB, CWM_LDMC, STN, and MBN, showed a higher slope and R-square with high EMF. In particular, the slope of AI, AGB, SWC, and MBN with high EMF was more than twice the slope of low EMF (0.008–0.016, 0.001–0.003, 0.603–3.482, and 0.002–0.004). Other factors such as ATP, SR, CWM_LP, CWM_H, and STP expressed an opposite relationship of different EMFs (Figure 3; Table 1).

**Table 1:** Results of the general linear regressions among low and high ecosystem multifunctionality (low EMF and high EMF) and influencing factors.

Observed variables	Low EMF				High EMF			
	R^2^	*p*	Slope	Intercept	R^2^	*p*	Slope	Intercept
GSL	0.004	*p* >0.05	−0.037	−0.170	0.018	*p* >0.05	−0.035	3.165
ATP	0.120	*p* <0.01	0.001	−0.627	0.010	*p* >0.05	0.000	0.482
AMT	0.017	*p* >0.05	−0.015	−0.394	0.190	*p* <0.01	−0.134	1.035
AI	0.100	*p* <0.01	0.008	−0.584	0.120	*p* <0.05	0.016	0.037
AGB	0.060	*p* <0.05	0.001	−0.495	0.130	*p* <0.01	0.003	0.197
BGB	0.120	*p* <0.01	0.032	−0.473	0.430	*p* <0.01	0.046	0.364
SR	0.020	*p* >0.05	0.008	−0.487	0.010	*p* >0.05	0.009	0.543
CWM_LN	0.007	*p* >0.05	0.003	−0.475	0.006	*p* >0.05	0.014	0.552
CWM_LP	0.087	*p* <0.05	0.085	−0.528	0.002	*p* >0.05	0.066	0.610
CWM_LDMC	0.050	*p* >0.05	0.002	−0.496	0.320	*p* <0.01	0.011	0.004
CWM_H	0.058	*p* <0.05	−0.009	−0.371	0.026	*p* >0.05	0.018	0.556
SWC	0.017	*p* >0.05	0.603	−0.483	0.569	*p* <0.01	3.482	−0.276
SBD	0.2395	*p* <0.01	−0.493	0.229	0.604	*p* <0.01	−1.956	2.390
SAP	0.182	*p* <0.01	0.055	−0.546	0.339	*p* <0.01	0.206	−0.015
SAN	0.168	*p* <0.01	0.002	−0.569	0.634	*p* <0.01	0.004	−0.346
SOC	0.360	*p* <0.01	0.017	−0.611	0.630	*p* <0.01	0.016	−0.078
STP	0.256	*p* <0.01	0.516	−0.602	0.274	*p* <0.01	0.969	−0.060
STN	0.430	*p* <0.01	0.229	−0.630	0.690	*p* <0.01	0.338	−0.306
MBC	0.210	*p* <0.01	0.001	−0.572	0.600	*p* <0.01	0.001	−0.122
MBN	0.090	*p* <0.05	0.002	−0.532	0.570	*p* <0.01	0.004	−0.351

GSL, the start of the growing season to the peak of the growing season; ATP, annual total precipitation; AMT, annual mean temperature; AI, aridity index; AGB, aboveground biomass; BGB, belowground biomass; SR, species richness; CWM_LN, community-weighted mean value of leaf nitrogen; CWM_LP, community-weighted mean value of leaf phosphorus; CWM_LDMC, community-weighted mean value of leaf dry matter content; CWM_H, community-weighted mean value of plant height; SWC, soil water content; SBD, soil bulk density; SAP, soil available phosphorus; SAN, soil available nitrogen; SOC, soil organic carbon; STP, soil total phosphorus; SAN; MBC, microbial biomass carbon; MBN, microbial biomass nitrogen.

**Figure 4:**
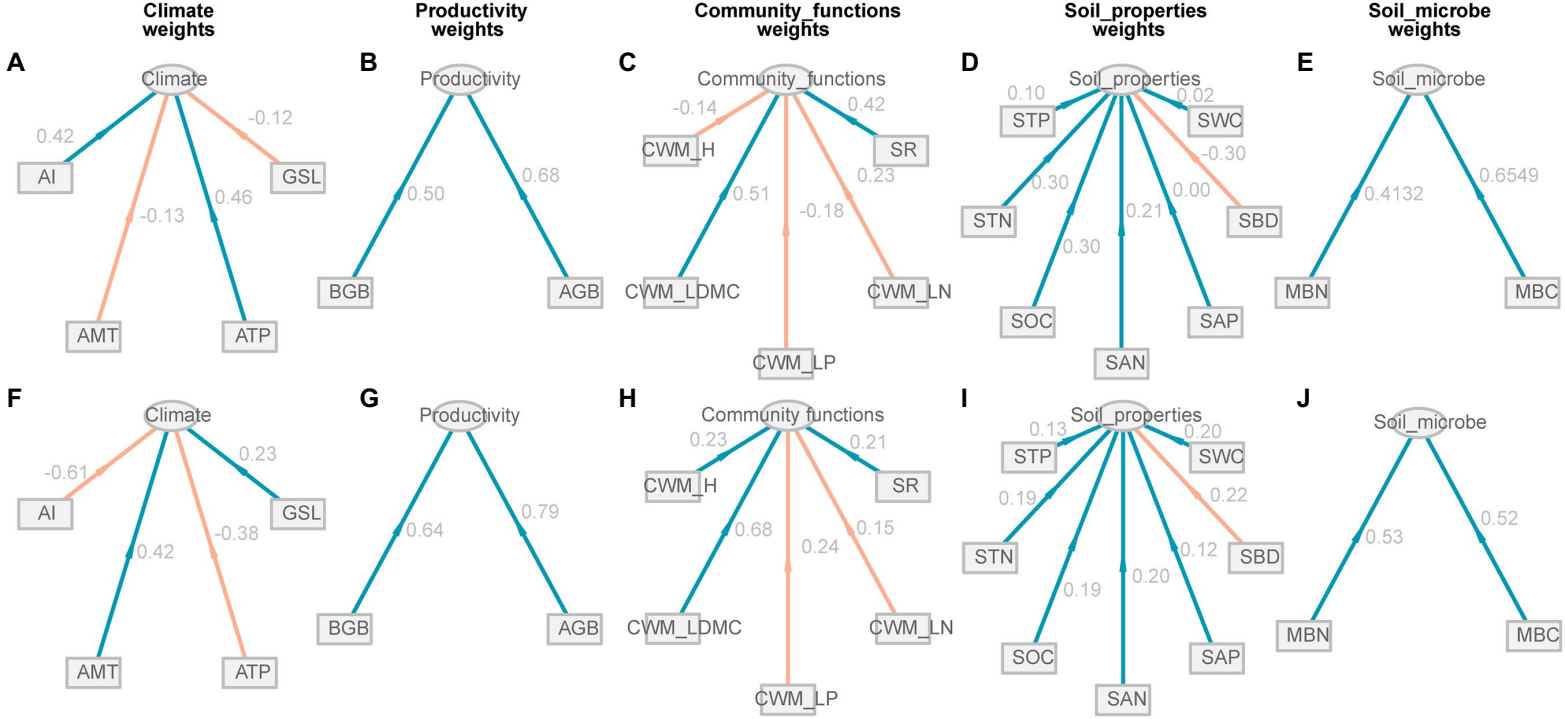
Relationship between observed variables and potential variables of climate, productivity, community functions, soil properties, and soil microbial factors. **(A–E)** Low ecosystem multifunctionality conditions. **(F–J)** High ecosystem multifunctionality conditions. The value next to the line represents the path coefficient between observed variables and potential variables. Red lines represent the negative weights, and green lines represent the positive weights. GSL, the start of growing season to the peak of growing season; ATP, annual total precipitation; AMT, annual mean temperature; AI, aridity index; AGB, aboveground biomass; BGB, belowground biomass; SR, species richness; CWM_LN, community-weighted mean value of leaf nitrogen; CWM_LP, community-weighted mean value of leaf phosphorus; CWM_LDMC, community-weighted mean value of leaf dry matter content; CWM_H, community-weighted mean value of plant height; SWC, soil water content; SBD, soil bulk density; SAP, soil available phosphorus; SAN, soil available nitrogen; SOC, soil organic carbon; STP, soil total phosphorus; MBC, microbial biomass carbon; MBN, microbial biomass nitrogen.

All environmental factors were divided into five potential variables, i.e., climate, productivity, community functions, soil properties, and soil microbe, under low EMF and high EMF conditions. For climate, positive weights were expressed by ATP (0.46) and AI (0.42) under low EMF conditions, but negative weights were expressed with the weight value of −0.38 and−0.61 under high EMF conditions, respectively (Figures 4A,F). However, productivity and soil microbe showed similar characteristics across different patterns (Figures 4B,E,G,J). CWM_H has a negative weight (−0.14) to community functions under low EMF conditions, but for a positive weight (0.23) under high EMF conditions (Figures 4C,H). Except for SBD, other factors have a positive contribution to the group of soil properties under different patterns (Figures 4D,I).

**Figure 5:**
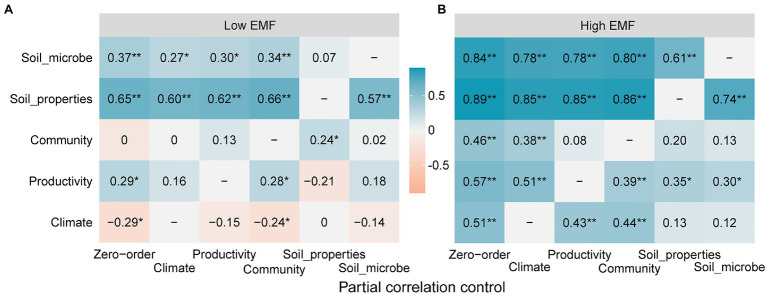
Partial correlations among potential variables of climate, productivity, community functions, soil properties, soil microbial factors, and ecosystem multifunctionality (EMF) under **(A)** low EMF and **(B)** high EMF conditions, respectively. The value in the square represents the value of the correlation coefficient between potential variables from ordinate and ecosystem multifunctionality after eliminating the influence of abscissa variables. Zero-order represents the correlation coefficient between potential variables and EMF. * and ** show the significant correlations at 0.05 and 0.01 levels.

According to the partial correlations among potential variables and low EMF, soil properties contributed most to low EMF. Particularly, after eliminating their influence, the partial correlation coefficients of soil microbe decreased from 0.37 to 0.07, and productivity decreased from 0.29 to −0.21 (Figure 5A). It was highlighted that community functions showed a huge increase from 0 to 0.24 after eliminating the influence from soil properties (Figure 5A). Additionally, no matter which potential variables are excluded, there is a negative correlation between climate and low EMF (Figure 5A). In high EMF conditions, soil microbe contributed most to EMF, which resulted in a reduction of partial correlation coefficients about climate (from 0.51 to 0.12), productivity (from 0.57 to 0.30), community functions (from 0.46 to 0.12), and soil properties (from 0.89 to 0.74; Figure 5B).

### SEM Mining the EMF Links to Environmental Factors

**Figure 6:**
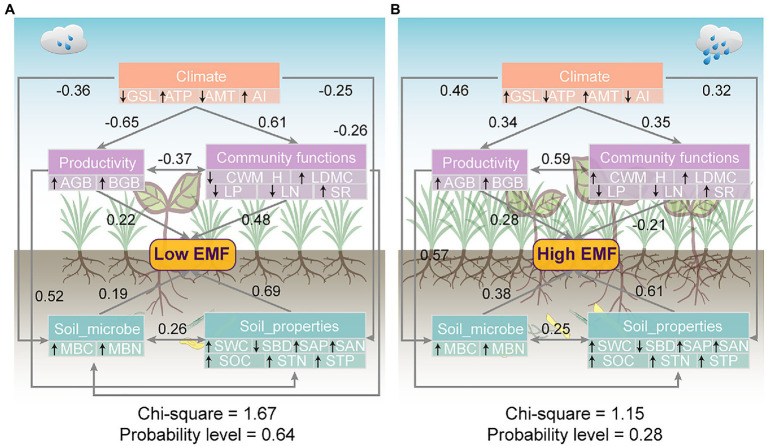
Mechanisms for the patterns of **(A)** low ecosystem multifunctionality and **(B)** high ecosystem multifunctionality. Structure equation model examining the standard total effects of climate, productivity, community functions, soil properties, and soil microbial factors on EMF. Given are the standardized total path coefficients. Gray arrows represent significantly paths (*p*<0.05). Black arrows pointing up or down in double-layer rectangles represent the positive or negative relationships between the observed variables and potential variables.

Path analyses demonstrated that climate generated significant negative effects on productivity, soil properties, and soil microbe with the standard total effects of −0.65, −0.25, and−0.36, respectively, under low EMF conditions (Figure 6A). EMF was determined by productivity, community functions, soil microbe, and soil properties with standard total effect values of 0.22, 0.48, 0.19, and 0.69, respectively (Figure 6A). In contrast, climate exerted significant positive effects on productivity, community functions, soil microbe, and soil properties with the standard total effects of 0.34, 0.35, 0.46, and 0.32, respectively, under high EMF conditions (Figure 6B). There were significant positive effects of productivity, soil microbe, and soil properties on EMF (effect coefficients are 0.28, 0.38, and 0.61, respectively; Figure 6B). Nevertheless, community functions had significant negative effects on high EMF with an effect coefficient of −0.21 (Figure 6B).

## Discussion

### Drivers of Low and High EMF Patterns

The relationship between EMF and its drivers may reflect the differences in the life histories of habitats, which are closely tied to above- and belowground communities (Jing et al., 2015). Our results emphasized that the abiotic and biotic environmental factors influenced EMF on the Tibetan Plateau (Figure 2). For the influence of climate, our results highlighted the important role of precipitation under low EMF conditions (Figures 2, 3; Table 1). Meanwhile, the AI index, which could reflect functions that drive the responses of plant nutrients to soils, plants, and eutrophication (Anderson et al., 2018), was positively correlated with high EMF with a higher slope than with low EMF (Figure 3). The plant biomass and species richness of alpine grassland generally increased from northwestern to southeastern Tibetan Plateau, which was closely related to the water–heat pattern (Zong et al., 2019; Sun et al., 2020b), and connected with the higher sensitivity response of low EMF. As for community functions, productivity, and soil properties, for example, AGB, CWM_LDMC, and SOC shaped EMF with higher sensitivity response under high EMF conditions (Figure 3; Table 1). Previous studies reported similar results on the relationships between biomass, soil nutrients, and EMF (Jing et al., 2015), which showed that, through the driving role of precipitation and soil moisture, higher water availability contributes to higher primary productivity and higher nutrient availability (Ma et al., 2010; Yang et al., 2010b; Sun et al., 2021b), leading to an increase in EMF. Besides, the role of soil microbial factors in driving different EMF patterns may be reflected by high correlations and sensitivity (Figures 2, 3). In grassland ecosystems, heterotrophic microbes usually accommodate with the suitable nutrient environment, which can promote a series of biochemical reactions belowground, and are more sensitive to the variability of soil nutrients (Sistla and Schimel, 2012). In addition, the change of microbial metabolic characteristics is affected by the synergistic effect of plants and soil. Under the facilitation of soil carbon and nitrogen, soil microbial diversity increases, which shows the effect of microbial biomass on high EMF (Wang et al., 2021). To illustrate the mechanism deeply, how the belowground community affects the different EMFs needs to be further explored.

### Mechanism of Low EMF

In our study, the partial correlation of community functions showed a shift on low EMF (Figure 4). Through the driver of the climate, SEM indicated a higher effect coefficient of community functions (0.48) on low EMF than soil microbes (0.19; Figure 6A). The key drivers of climatic factors such as ATP have been proven to shape the positive relationship between biodiversity and EMF (Ma et al., 2010). On the one hand, low EMF environments are usually accompanied by drought, the increase in precipitation could increase species richness and further induces the increase in EMF (Xu et al., 2018). On the other hand, researchers revealed that precipitation amendment had indirect positive effects on community functions and may play a key role in improving ecosystem stability (Falster and Westoby, 2003). Hence, our results showed high weights of CWM_LDMC (0.51) and SR (0.42), which led to low EMF (Figures 4, 6A). A previous study revealed that by increasing CWM_H, a vital trait that impacts plant carbon sequestration capacity, community productivity could have an indirect positive effect on EMF (Moles et al., 2009). However, our results showed a negative effect of plant height on low EMF, which is related to the hydraulic limitation hypothesis that with increasing plant height, the difficulty in supplying leaves with water and nutrition also increases (Moles et al., 2009), especially under poor environmental conditions. Moreover, productivity has direct effects (0.22) on low EMF (Figure 6A), which indicates that the positive effects of productivity, especially belowground productivity, on multifunctionality may be the main mediated function through increased community traits under a relatively suitable environment. Consequently, our results showed that under the low EMF environment, the primary task of the plant community may be to survive (Wang et al., 2020b).

Several studies have demonstrated that soil microbial communities play a key role in driving the resistance of EMF (Bunge et al., 2006; Wagg et al., 2014; Delgadobaquerizo et al., 2017). However, in this study, our results suggested that soil microbes had minor effects at EMF within low patterns (0.19; Figure 6) on the Tibetan Plateau scale. Consistent with several other studies in grassland ecosystems, an increase in soil nitrogen availability could increase plant community productivity and EMF (Jing et al., 2015; Demalach et al., 2017). Nitrogen is imperative for the production of structural proteins as building portions of plant tissues (Gan et al., 2010). Accordingly, nitrogen has been proven to accelerate net carbon sequestration and contribute to increased community plant productivity across grassland ecosystems (Niu et al., 2010; Xu et al., 2018), further improving low EMF. Nevertheless, the biochemical processes of the ecosystem are often limited by the availability of organic carbon, especially in arid and semiarid habitats (Bapiri et al., 2010; Liu et al., 2020). Hence, the availability of soil nutrients may be the main explanation for the low effects of microbial factors on low EMF (Figure 6A). Summarily, based on the above driven by climate factors, the plant community plays a more crucial role in low EMF.

### Mechanism of High EMF

In contrast to the low EMF pattern, our findings suggest that AMT showed stronger positive effects on high EMF than precipitation, with weight values of 0.43 (Figures 4, 6). Due to sufficient precipitation conditions, especially in an alpine meadow, the increase in temperature could promote community diversity and soil nutrient accumulation (Sun and Wang, 2016). For community functions, plant traits can reflect the adaptability of plants to environmental changes and the nutrient utilization efficiency of plants (Thompson et al., 1997). However, in this study, community functions showed negative effects on high EMF with a path coefficient of −0.21 (Figure 6A), which may be linked with low leaf nutrient turnover in higher soil nutrient environments (Sun et al., 2021a). Previous studies identified specific microbial taxa that are probably major drivers of EMF, which is also supported by many experiments showing that the total abundance of microbes controls particular functions (Delgadobaquerizo et al., 2017). As highlighted, MBC and MBN were indicators of nutrient availability that included quickly available sources for microbial activity and were strongly linked to the activity of the soil microbial community (Bastida et al., 2016). Therefore, it is possible that under low EMF conditions, the activity of microorganisms is limited by soil nutrients, while under high EMF conditions, microorganisms can play an important and positive role, and the final results of SEM show an effect of microbe groups on high EMF (Figure 6). In addition, high EMF is often connected with high plant diversity, and higher levels of carbon inputs to the soil linked to more diverse plant communities result in more active, more abundant, and more diverse soil microbial communities (Lange et al., 2015). Therefore, when the soil resources are sufficient to maintain the growth of plants, the increase in microorganism activity and biomass plays a major role in maintaining high EMF.

### Importance and Uncertainties

Most of the regions in the world are undergoing climate warming and species diversity loss, especially on the Tibetan Plateau (Rockstrom et al., 2009; Zhou et al., 2017). Increasing efforts have been conducted to explore the internal mechanism and consequences for ecosystem stability (Jing et al., 2015). Three main aspects of our study distinguish it from the few previous studies on this topic. First, few studies have considered the shifting role of plant community functions and microbe factors, which could change the effects of several factors on EMF, and our study examines both reliability and sensitivity over a wide range. Second, we compare the response of EMF to abiotic and biotic factors under high and low EMF patterns. Third, our study builds a model for above- and belowground and demonstrates that plant and microbial factors mediate the shift of EMF from low to high patterns under the influence of precipitation.

Although the level of high and low EMF is distinguished, the high and low conditions of EMF are only a relative concept due to the method of measurement of the standards. Besides, due to the limitation of data, we used only MBC and MBN to represent microbial factors, which might not fully reflect microbial indicators.

## Conclusion

Our work indicates that high EMF may show a higher sensitivity response to climate, productivity, community functions, soil properties, and soil microbial factors. Moreover, our results suggest that plants and microbes mediated the shift of low to high EMF through the comprehensive effect of climate and environmental factors. As climate change and biodiversity of above- and belowground are lost from ecosystems, predicting how ecosystems will function in the future will require more experiments and observations.

## Data Availability Statement

The original contributions presented in the study are included in the article/supplementary material, further inquiries can be directed to the corresponding authors.

## Author Contributions

YiW, FX, and ZZ: conceptualization and software. YiW and ML: methodology, investigation, and writing – original draft preparation. ML: validation and data curation. YiW: formal analysis and resources. YiW, ML, YC, TZ, XL, BY, YaW, LZ, XN, FX, ZZ, and JS: writing – review and editing. All authors contributed to the article and approved the submitted version.

## Funding

This research was supported by “Construction Project of Characteristic Specialty and Experimental Training Teaching Base (Center) of Guangxi Undergraduate Colleges and Universities in 2018-2020 [Guijiao Higher Education (2018) 52],” Second Tibetan Plateau Scientific Expedition and Research (No. 2019QZKK0405), and National Natural Science Foundation of China (No. 31901198).

## Conflict of Interest

The authors declare that the research was conducted in the absence of any commercial or financial relationships that could be construed as a potential conflict of interest.

## Publisher’s Note

All claims expressed in this article are solely those of the authors and do not necessarily represent those of their affiliated organizations, or those of the publisher, the editors and the reviewers. Any product that may be evaluated in this article, or claim that may be made by its manufacturer, is not guaranteed or endorsed by the publisher.

## Footnotes

^1^ http://data.cma.cn/
